# Biochar and Organic Fertilizer Co-Application Enhances Soil Carbon Priming, Increasing CO_2_ Fluxes in Two Contrasting Arable Soils

**DOI:** 10.3390/ma16216950

**Published:** 2023-10-30

**Authors:** Magdalena Bednik, Agnieszka Medyńska-Juraszek, Irmina Ćwieląg-Piasecka

**Affiliations:** Institute of Soil Science, Plant Nutrition and Environmental Protection, Wrocław University of Environmental and Life Sciences, Grunwaldzka 53 St., 50-375 Wrocław, Poland; magdalena.bednik@upwr.edu.pl (M.B.); irmina.cwielag-piasecka@upwr.edu.pl (I.Ć.-P.)

**Keywords:** biochar, soil respiration, incubation experiment, CO_2_ efflux, carbon sequestration

## Abstract

Biochar soil amendments, along with non-tillage agriculture, are often proposed as a strategy for carbon sequestration. It is still questionable how the quality of biochar might influence the priming effect on soil organic matter and whether the addition of unprocessed organic amendments will affect biochar stability. In the study, six different biochars and three exogenous organic matter sources were added to two distinct arable soils. CO_2_ emission was monitored for 100 days of incubation and CO_2_ flux was estimated. Results showed that biochar increased soil CO_2_ fluxes. The highest peaks, up to 162 µg C-CO_2_ h^−1^ 100 g^−1^, were recorded in treatments with food waste biochars, suggesting that they serve as a source of easily available carbon to soil microbes. Co-application of raw organic materials (manure and fresh clover biomass) enhanced CO_2_ emission and carbon losses, especially in sandy soil, where 0.85–1.1% of total carbon was lost in the short-term experiment. Biochar properties and content of labile C can stimulate CO_2_ emission; however, in a long-term period, this contribution is negligible. The findings of our study showed that more attention should be paid to priming effects caused by the addition of exogenous organic matter when applied to biochar-amended soils.

## 1. Introduction

Some intensive agriculture strategies contribute to the increase of greenhouse gases (GHG) emissions, and biochar (BC) has been widely recommended as a soil amendment, moderating global climate change. Produced by the thermochemical conversion of organic residues in oxygen-limited conditions, biochar is highly resistant to degradation due to its recalcitrant nature [1,2]. The addition of biochar to soil alters physicochemical properties, e.g., porosity, bulk density, pH, carbon (C), and nitrogen (N) content or water holding capacity, which impact soil CO_2_ emissions [3,4,5]. BCs obtained from various feedstock, under different temperature regimes of pyrolysis, have various properties [6] and their effects after incorporation into the soil may greatly vary with local environmental conditions and cultivation systems [7,8]. In general, biochars produced from plant biomass, e.g., straw or wood, are rich in recalcitrant C forms and are able to sequester more C in soil in comparison with biochars derived from animal manures [9] or food wastes [10]. Biochar application to soil may increase carbon sequestration due to the inputs of recalcitrant organic C [6,11,12]; however, the effect of biochar application on soil GHG emissions is questionable. Results presented in a meta-analysis show that biochar application significantly increased soil CO_2_ fluxes by 22.14%, thus contributing to the global warming potential [13]. The mechanisms behind this process are still not well understood. The exogenous inputs of labile C sources such as fresh plant residues or dissolved organic carbon (DOC) from pyrogenic organic matters (POMs) to soil induce a positive priming effect with increasing CO_2_ emission [14,15]. On the other hand, some studies reported negative priming and suppression of soil CO_2_ emission due to reduced enzymatic activity and the precipitation of CO_2_ on the biochar surface [16]. Furthermore, the direction of priming effects may change over time [17]. To assess the possible contribution of biochar to GHG mitigation and its stability in soil, it is necessary to include many factors that might affect biochar behavior in soil under different climatic conditions and cultivation systems [18,19,20]. The potential of biochar for long-term carbon sequestration is affected by numerous factors, such as biochar and soil properties, soil organic matter quality, or even temperature and soil moisture level, that may affect microbial activity and thus enhance or prevent carbon losses [21,22]. It is also important to answer the question of whether all biochars contribute to the GHG mitigation process equally, or if maybe more attention should be paid to the final product in terms of finding a proper material for effective CO_2_ emission mitigation from cultivated soils. As non-tillage and organic farming strategies to increase the carbon sink in agricultural soils are receiving a lot of attention, biochar co-application with sustainable tillage practices might be a proper approach to supporting greenhouse gases emission mitigation [23,24]. The knowledge about the effects of the co-application of biochar, raw crop residues, and organic fertilizers, e.g., manure and compost on CO_2_ emissions from arable soils, is limited.

An increasing number of studies have demonstrated that soil organic carbon (SOC) decomposition can be influenced by exogenous organic C (EXOC) input through the priming effect. For example, Sun et al. [25] claimed that the addition of EXOC significantly enhanced native SOC decomposition with the highest value in cropland soils, which contributes greatly to CO_2_ emissions. Our study gives insight into the state of knowledge about biochar CO_2_ mitigation potential in soil, answering the question of whether the process of carbon sequestration by biochar can be disturbed by the application of other exogenous organic matter. Based on the literature, it can be hypothesized that labile organic matter (LOM) from cover crop residues or organic fertilizers, e.g., manures or compost, may change the C-sequestration potential in biochar-amended soil, as both types of C sources will contribute to the SOC priming effect. This may induce changes in the native mineralization process of organic matter, which, in turn, will increase or decrease CO_2_ flux from soil. Moreover, the presence of raw organic residues and labile C fractions may influence the biochar mineralization rate and this can be indicated by CO_2_ emissions during respiration processes [26]. Previous studies have mainly examined biochar produced from forestry and agricultural wastes. As a novel approach, in this study, the recalcitrance of conventional straw and wood biochar is compared with biochar produced from kitchen wastes—mainly food scraps, fruit and vegetable peels, and all the wastes selectively collected for the composting process. Food waste conversion to biochar has been widely studied as a method to sequester carbon and mitigate substantial greenhouse gas emissions, as signed in the United States 2030 Food Loss and Reduction Goal [27]. Our previous study showed that food waste biochar contains more labile carbon compounds prone to oxidation and thus can contribute to the process of CO_2_ emission from BC-amended soil or enhance soil organic matter (SOM) mineralization [10], and both processes can be monitored by measuring CO_2_ efflux from soil. Biochar recalcitrance is expected to last hundreds of years [1,2], but residence calculations in most of the studies do not take into account the carbon loss due to enhanced mineralization of biochar in the presence of raw organic matter delivered to soil with organic fertilization on non-tillage cultivation strategies.

The paper assesses the effects of biochar on CO_2_ efflux from soils amended with biochars derived from different waste materials. It also verifies the questionable effect of exogenous raw organic matter on biochar recalcitrance under conditions imitating non-tillage soil cultivation, promoted as a sustainable method of soil conservation and reduction of agricultural impact on GHG emission.

## 2. Materials and Methods

### 2.1. Incubation Experiment Setup

The incubation experiment was carried out with two different soil types, six biochars, and three types of additional organic matter mixed with soil. Both soils used in this study are common in Central Europe and the main intended difference between them is the texture—silt loam (SiL) and loamy sand (SA) (Table 1). Samples were collected from the topsoil (0–25 cm) layer of arable land in two locations close to Trzebnica, Poland (51°15′46.8″ N 17°06′13.3″ E and 51°24′13.2″ N 17°06′31.6″ E). Prior to the incubation experiment, moist soil samples were stored in closed containers in the refrigerator at 4 °C to maintain soil biological activity. Six different feedstocks commonly produced in urban areas and farmlands were chosen for biochar production: kitchen wastes (BC1), cut grass (BC2), coffee grounds (BC3), wheat straw (BC4), sunflower husks (BC5), and beech wood chips (BC6). All the feedstocks are accepted as permissible biomasses for biochar production in Europe [28]. Before the pyrolysis, feedstock materials were air-dried and stored at ambient air humidity. The production of biochars was performed in September 2020 at Wroclaw University of Technology. Pyrolysis was conducted in the nitrogen atmosphere at 550 °C and the conditions remained constant for every type of feedstock. The duration of the process was 60 min for each biomass. Organic matter applied in this experiment included compost (CO), cattle manure (MA), and fresh legume biomass (LE). Compost was produced in a home composter located in Wroclaw, Poland, from kitchen waste (vegetable and fruit peels) and garden waste (cut grass, leaves, and small twigs). Dried cow manure in the form of granules was purchased from Fertigo fertilizer supplier (Suchy Las, Poland). Legume biomass consisting of whole plants of white and red clover (*Trifolium repens* L., *Trifolium pratense* L.) was collected from green areas in Wrocław, Poland.

Biochars, compost, and manure were air-dried and ground in a soil mill to obtain particle sizes <2 mm. Fresh legume biomass was carefully washed with distilled water to avoid the contamination of incubation systems and cut into pieces <2 mm with scissors to obtain materials with uniform fraction sizes. Biochars and organic materials with particle sizes <2 mm were mixed thoroughly with the soil in the following proportions: biochars 2% (*v*/*w*) (corresponding to additions of 0.565–0.915 t ha^−1^ depending on the biochar’s bulk density, assuming the thickness of the plowing layer of 25 cm and a soil density of 1.50 g cm^−1^) and organic matter 1% (*w*/*w*) (corresponding to the dose of 37.5 t ha^−1^). Then, 100 g of each mixed sample was placed in a 550 mL glass vessel. All treatments are summarized in Table 2. Vessels were incubated in a place protected from direct sunlight at a constant air temperature of 22 °C. They were left open most of the time to allow soil respiration, with the possibility to close tightly if needed. The moisture of the incubated material was maintained at approx. 20% by weight by watering with distilled water when necessary.

### 2.2. Analysis of Substrates

To determine the standard characteristics of the substrates, samples of the soils, biochars, compost, and manure were air-dried, sieved with 2 mm mesh, and further prepared following the specific methodologies of analyses. The pH was determined in H_2_O in a 1:5 suspension (*v*/*v*) using a pH meter (Mettler-Toledo, Graifensee, Switzerland). For soil samples, particle size distribution was measured using the mesh and hydrometer method, and the content of calcium carbonates as an equivalent was determined using the Scheibler apparatus (according to DIN 18129, ISO 10,693 method), an approach frequently applied in Poland to determine CaCO_3_ content in soil samples [29,30]. Cation exchange capacity was measured by an MP-AES 4200 Spectrometer (Agilent Technologies, Santa Clara, CA, USA) after extraction with 1 M ammonium acetate. For biochars, a modification of the method was used, based on rinsing the samples with isopropanol, as proposed by Munera-Echeverri et al. [31]. Total organic carbon and total nitrogen were measured on an enviro TOC/TN analyzer (Elementar, Langenselbold, Germany). Ash content was calculated based on mass loss after sample combustion in a muffle furnace at 550 °C (Czylok, Jastrzębie Zdrój, Poland). Labile carbon fractions in biochars were examined in a previous study [10]. The characteristics of the soils, biochars, and organic materials used as substrates for the experiment are summarized in Table 2.

### 2.3. Respiration Measurements

**Figure 1:**
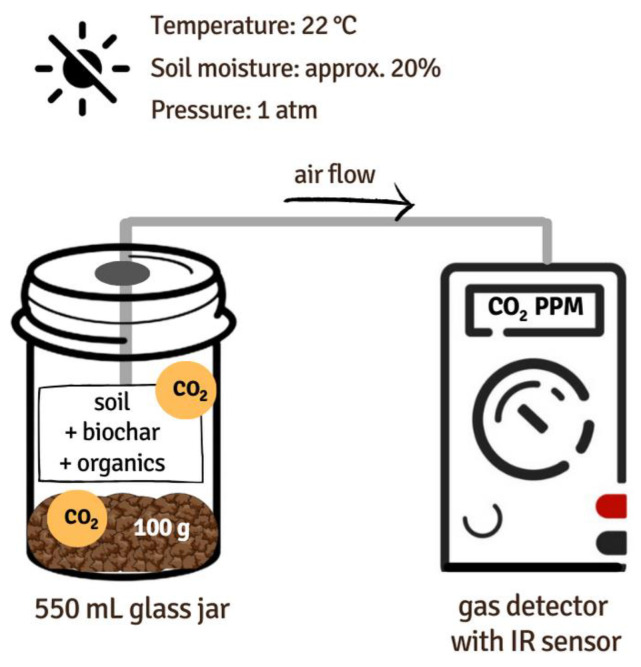
Scheme of the soil respiration measurements.

Soil respiration was measured during the incubation as the amount of CO_2_ released by the unit of soil + treatment in the unit of time. To determine this value, a portable gas detector with an infrared (IR) sensor dedicated to CO_2_ concentration measurements (GasHunter II, Alter S.A., Tarnowo Podgórne, Poland) was used. The measuring range of the device was 0–5000 ppm with a resolution of 50 ppm. The assumption was that after closing the vessels, the concentration of CO_2_ would increase over time only as an effect of soil respiration. Each sampling began with the sealing of the jars for one hour directly before CO_2_ concentration measurements, to allow the gas to accumulate. Then, carbon dioxide levels were measured in the air inside the vessel by inserting the probe with the pump through a dedicated valve in the cap and collecting the sample for 60 s (please see the scheme below—Figure 1). After the measurement, jars were left open to allow equilibration of O_2_ and CO_2_ levels with the atmosphere and ensure sufficient soil aeration.

Measurements were conducted in three replicates and the final value is an average. The zero value as a reference point was the CO_2_ concentration in the air in the laboratory. Measurements were carried out at 1, 3, 5, 7, 14, 35, 42, 55, 70, 84, and 98 days of incubation. After this time, the CO_2_ was constant and at a very low rate, close to the detection limit of the device. The temperature and air humidity in the room were constant during the measurements (22 °C, humidity approx. 50%). To control these conditions, automatic sensors of air parameters were used and conditions in the incubation room were adjusted by air-conditioning if needed.

Values recorded by the CO_2_ sensor were displayed in ppm, therefore it was necessary to perform some calculations. We adapted the protocol proposed by Fierer [32], based on the universal gas law to convert ppm CO_2_ to C-CO_2_ [µg]. We assumed that the pressure and temperature were constant during all the measurements (1 atm and 22 °C), and the volume of free space in the vessel was 490 mL (the remaining volume from 550 mL was taken up by soil). To calculate the number of moles of air in the vessel (*n*) the modified ideal gas law was applied:

$$n=\frac{pV}{RT}$$

where *V* = volume of air in the vessel (490 mL), *p* = pressure (1 atm), *R* = const. [82.05 mL atm mol^−1^ K^−1^], *T* = temperature in K = 273 + °C [273 + 22 = 293 K]. According to the calculations, each vessel contained 20.38 mmol of air. To determine the exact amount of C-CO_2_ released by the incubated mixture, the following equation was applied on the basis of laboratory protocol by Fierer [32].

$$\mu g\;C-{CO}_{2}=mmol\;air\times ppm\;{CO}_{2}\times 10^{-3}\frac{mol}{mmol}\times 12\frac{\mu g\;C}{\mu mol\;C}$$

The calculated values relate to µg of C-CO_2_ released by 100 g of soil in one hour.

Graphs and figures were prepared with GraphPad Prism 5 Software for Windows (GraphPad Software Inc., San Diego, CA, USA). Calculations of results were performed using MS Excel Professional Plus 2019 Software (Microsoft, Redmond, WA, USA) and GraphPad Prism 5 Software for Windows.

### 2.4. Carbon Loss Estimation

Carbon loss was balanced as a percentage of carbon released during the respiration measurements in relation to the whole carbon pool present in incubated vessels, which originated from native soil organic matter, biochars, and organic amendments (compost, manure, or legume biomass). The results of cumulative respiration were calculated to obtain the mass of released carbon. Carbon content in soil, biochar, and organic amendment was determined before the experiment in dry substrates. Then, the amount of C introduced with biochar and organic amendment required the following calculations.

For biochars, calculations were based on carbon content in dry substrates and bulk density of biochars using the formula:

BCC=ρ×V×%C [g]

where *BCC* = carbon originating from biochars, *ρ* = bulk density of biochars [g cm^−3^], *V* = amount of biochar in vessel (2 cm^3^), %*C* = carbon content in biochars.

For compost, manure, and legume biomass, calculations included dry mass and carbon content in the substrate:

COA=m×d.m.×%C [g]

where *COA* = carbon originating from organic amendment, *m* = mass of amendment in the vessel (1 g), *d.m*. = content of dry mass [%], %*C* = carbon content in dry mass of the amendment.

Then, the carbon pool introduced from organic amendments and biochars and present in soil was summarized to obtain the total C content in incubated vessels (g 100 g^−1^ soil). Total carbon content was compared with carbon losses during respiration, which allowed the expression of the loss as a percentage of the carbon pool. Results were summarized in Table S1—Supplementary Materials.

## 3. Results

### 3.1. Effect of Soil and Biochar Type on Respiration

**Figure 2:**
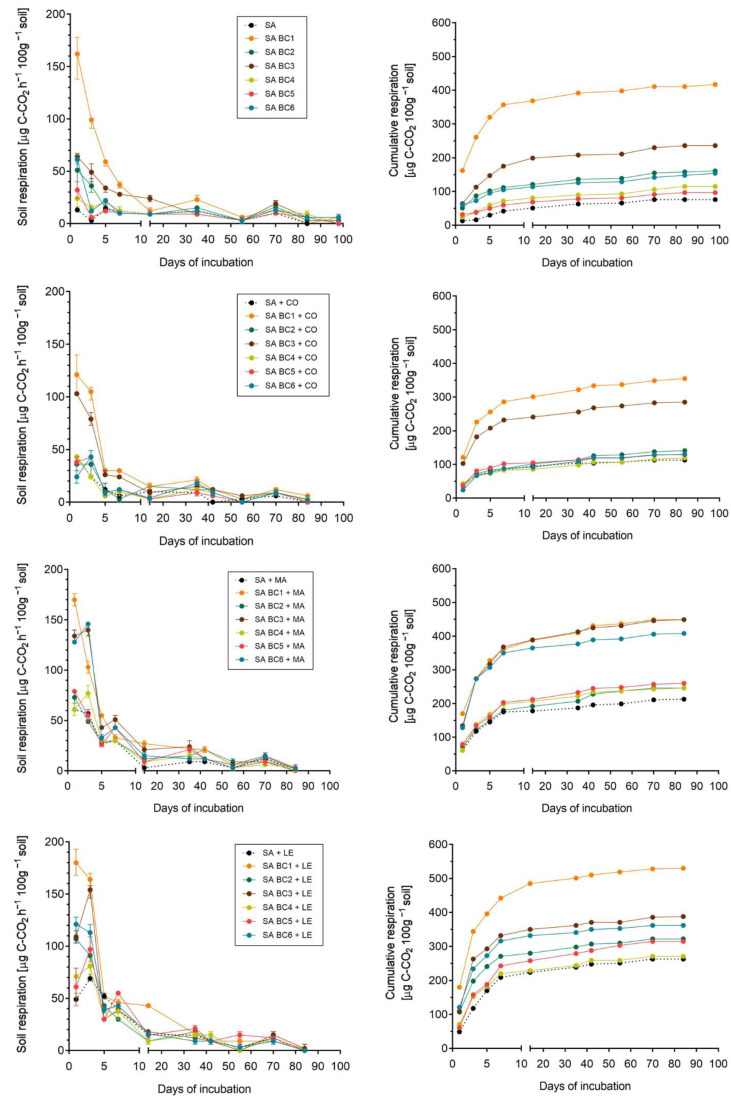
Respiration of sandy soil (SA) amended with biochars (BC1–BC6) and organic materials: compost (CO), cattle manure (MA), and legume biomass (LE). Point = mean, bars = minimum and maximum. BCs origins: BC1 = kitchen waste, BC2 = cut grass, BC3 = coffee grounds, BC4 = wheat straw, BC5 = sunflower husks, BC6 = beech wood chips.

**Figure 3:**
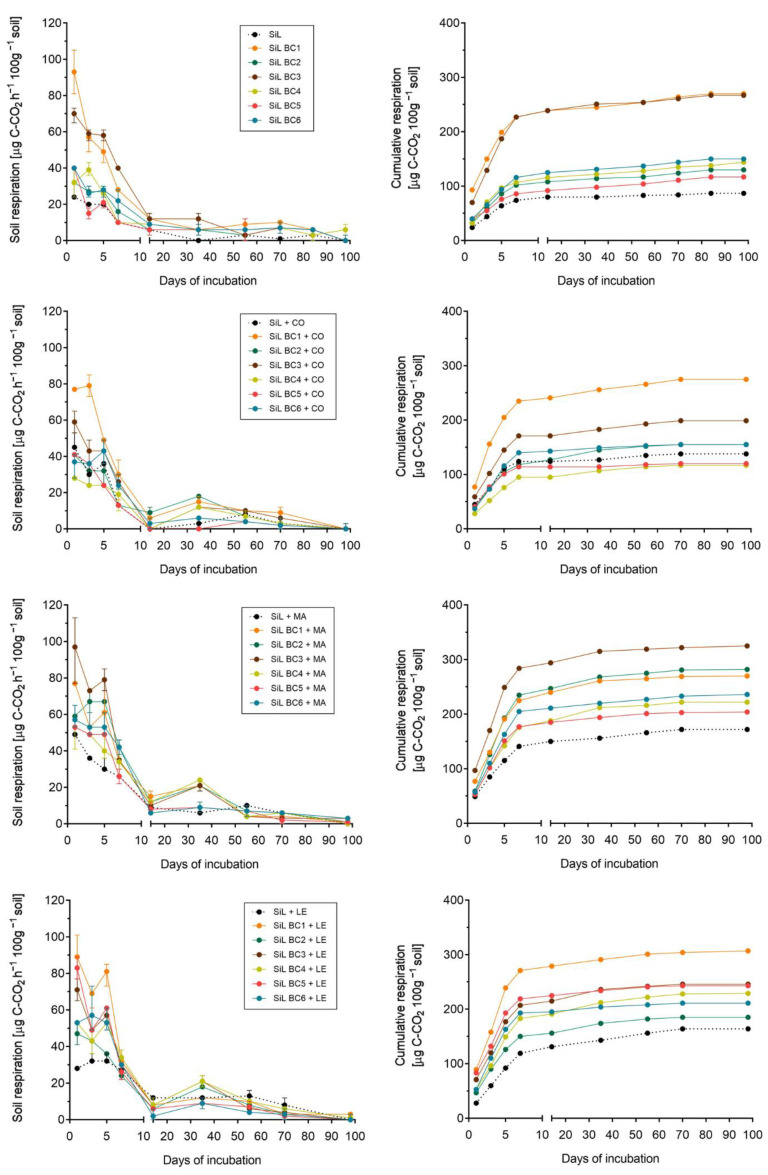
Respiration of silt loam (SiL) amended with biochars (BC1–BC6) and organic materials: compost (CO), cattle manure (MA), and legume biomass (LE). Point = mean, bars = minimum and maximum. BCs origins: BC1 = kitchen waste, BC2 = cut grass, BC3 = coffee grounds, BC4 = wheat straw, BC5 = sunflower husks, BC6 = beech wood chips.

During 100 days of incubation, in each variant, regardless of soil characteristics and type of biochar, the highest respiratory activity was indicated during the first 7 days. After the initial peaks and some fluctuations in respiration, strong decreases in CO_2_ release were noted and after about 10 days of the experiment, recorded values were definitely lower and stable. Despite similar trends over time (the highest CO_2_ evolution in the first week of incubation, followed by a sharp decrease and stabilization of the values), measured values differed between sandy (SA) and loamy (SiL) soil. Carbon dioxide evolution tended to be higher in SA compared with SiL amended with analogous doses and types of biochars (Figure 2 and Figure 3). The largest CO_2_ emission was from sandy soil with BC1, up to 162 µg C-CO_2_ h^−1^ 100 g^−1^ of soil (mean value), recorded on the 1st day of incubation with biochar made from food waste (Figure 2). Treatment with BC1 was also associated with the highest respiration in loamy soil (Figure 3). The second biochar that led to remarkably higher respiration rates in both soil types was derived from coffee grounds (BC3). The pattern between all the treatments reveals that the non-amended control soils had a lower respiration rate than samples incubated with biochars. Moreover, CO_2_ evolution from SiL tends to be lower than from sand mixed with the same biochar types, regardless of the fact that loamy soil had a higher initial carbon content (0.99 g 100 g^−1^ vs. 0.72 g 100 g^−1^ on SA) (Table 1).

Carbon losses were calculated on the basis of TOC in soil and amendments compared with the amount of carbon lost as C-CO_2_ during respiration. Although in none of the treatments calculated C depletion exceeded 1%, there were some clear differences between variants in the experiment. During 100 days of incubation, sandy soil (SA) amended only with biochars lost from 0.22% (SA + BC5) to 1.01% (SA + BC1) of the total organic carbon present in the incubated mixture, whereas silt loam (SiL) mixed with the same biochar types exhibited lower declines of C content in the range of 0.21% (SiL + BC5) to 0.52% (SiL + BC1) (Table S1—Supplementary Materials).

### 3.2. Effect of Exogenous Organic Matter on Soil Respiration

Considering variants with additional organic amendments—compost, manure, and legume biomass—higher CO_2_ evolution rates were obtained from soils treated with BCs + manure and legume biomass than with compost. For sandy soils, in variants with manure, respiration day-by-day reached up to 145–170 µg C-CO_2_ h^−1^ 100 g^−1^ in 3 out of 7 tested combinations, whereas legume biomass addition caused even higher CO_2_ release, with a maximum of 180 µg C-CO_2_ h^−1^ 100 g^−1^ (SA BC1 + LE). In addition, it was noted that SA + MA and SA + LE treatments showed values of respiration around 30–40 µg C-CO_2_ h^−1^ 100 g^−1^ for a longer time (approx. 30 days) than soils amended with compost or with biochars only (Figure 2). Treatments with SiL followed the same trend. As within the sandy soils, the respiration rate increased in silt loams amended with biochars and legume biomass or manure, whereas compost had a lesser effect on CO_2_ evolution. Moreover, organic amendments, especially legumes and manure, resulted in a longer persistence of high soil respiration values—after 14 days of incubation, the CO_2_ evolution in SiL + MA or SiL + LE treatment rates was still relatively high, around 20–40 µg C-CO_2_ h^−1^ 100 g^−1^ (Figure 3). Considering carbon loss percentage in biochar + organic amended soils, the addition of easily decomposable organic matter generally increased the percentage of carbon loss, especially in manure-treated soil with BC (up to 0.85% in SA BC1 + MA) and legume biomass (1.10% in SA BC1 + LE). The effect of compost on the dynamics of C losses during respiration was less evident, as the maximum reached 0.73% (SA BC1 + CO), whereas most values in compost-amended soil were 0.2–0.3%, both in sandy and silty soils (Table S1—Supplementary Materials).

To sum up, initial soil carbon status (native organic carbon pool) had no effect on observed CO_2_ efflux—respiration rate was even lower in SiL than in SA soil. Regardless of the soil type, BCs showed similar patterns of respiration among tested treatments. Carbon dioxide emissions were the highest directly after the application of the amendment, and the maximum values were observed for BCs rich in labile organic compounds, such as BC1 or BC3. This strongly suggests that the initial peak of CO_2_ evolution was a result of labile decomposition from biochars. Moreover, among tested exogenous organic matter sources, raw materials (legume biomass and manure) had a greater impact on C mineralization rates than biologically stable OM from compost, which was reflected in remarkably lower respiration in CO_2_-amended treatments compared with LE or MA. Generally, the trend in CO_2_ evolution due to the type of biochar is: BC1 > BC3 > BC6, BC2 > BC5, BC4, and due to the additional exogenous organic matter source, it is: legume biomass > manure > compost.

## 4. Discussion

The results of the study showed that under controlled environmental conditions, biochar amendments affected GHG emissions, increasing CO_2_ release from soils. The stimulating effects of biochar application on soil CO_2_ fluxes can be ascribed to higher labile C mineralization and inorganic C release from biochar [13]. Furthermore, biochar application supports labile soil organic carbon pools, enhancing microbial activity [33]. Microbial available C and nutrients in biochar are strongly correlated with the temperature of pyrolysis [1,34]; however, findings of our study support the thesis that biomass origin and properties of biochar, especially the content of labile C fractions, will contribute to the process of SOC mineralization, stimulating CO_2_ emission from soil. The addition of biochar with more labile C fractions, e.g., derived from kitchen wastes, contributed to the process of CO_2_ emission from soil more prominently than biochars derived from high lignocellulose biomass, e.g., wood chips or straw. This observation is in agreement with our previous findings described by Bednik et al. [10], where exact amounts of easily convertible carbon forms in biochars were calculated. A higher content of water-soluble carbohydrates (WSC) or dissolved organic carbon (DOC) and less aliphatic structure of biochars derived from kitchen wastes such as coffee grounds (BC3) or vegetable and fruit peels (BC1) serve as labile C sources for microbes when applied to soil. Similar patterns in BC mineralization were observed by Farrell et al. [35], showing that soil microbes rapidly utilize easily available carbon pools delivered with biochar in the forms of carbohydrates, dissolved organic carbon, or volatile solids, but also a wide range of other organic compounds. Further findings of our experiment included the activity of three carbon cycle enzymes along with dissolved organic carbon content in the incubated material, and a positive relationship between CO_2_ evolution, the content of the labile carbon fraction, and the activity of microorganisms was confirmed [36]. Results indicated that CO_2_ fluxes varied over time after biochar application, which is in agreement with the literature findings [37,38,39]. However, mechanisms involved in soil CO_2_ stimulation after biochar application may differ in the short term compared to long-term study. The effect of the breakdown of organic C and the release of DOC from biochar is the stimulation of CO_2_ emission from soil in a very short amount of time after biochar application (up to 7 days). After sources of readily available carbon are utilized, CO_2_ flux in biochar-amended soils was stable, however higher compared to un-amended soils. This confirms that biochar can cause a priming effect on native soil organic matter, but in the long term, the process of CO_2_ emission directly from biochar transformations becomes negligible, thus not contributing to GHG emission on a global scale [17,39]. It is known that young biochars contain a pool of labile carbon that is easily and rapidly convertible by soil microorganisms as a source of energy. In the long term, remaining complex and aromatic structures determine biochar’s resistance to decomposition processes [40]. The application of biochar to tested soils also affected carbon pools, causing carbon losses, probably due to disturbance of the soil environment (input of nutrients and labile carbon sources) [37]. Usually, SOC content increases in the short term are due to the application and incorporation of fresh and C-rich biochar into the soil. This initial exposition of fresh biochar leads to a high microbial response and the turnover of the labile C fractions, often referred to as a positive priming effect [41].

Carbon losses can be also correlated with soil properties and this phenomenon was observed in the study. According to Gross et al. [42] in their meta-analysis, biochar application to clay soils resulted in the highest SOC stock increase, followed by silty soils and loamy soils. The lowest increases were observed for sandy soils. In general, a higher clay mineral content in finer textured soils not only provides physical protection of SOC to enzymatic activity and thus turnover but also increases SOC stability in the form of aggregates [43]. The stability of SOC depends also on soil pH—positive priming is more common in acidic soils, mainly due to liming and co-metabolism being an effect of introduced nutrients and habitat, which promotes the activity of heterotrophic microbes, and that trend was observed in our research. Moreover, pH is important for soil CO_2_ release. Neutral or alkaline soils, represented by SiL in this study, are able to effectively adsorb CO_2_, which may lead to lower respiration values when compared to acidic soil, as was noticed [44]. The effects of biochar application on soil CO_2_ fluxes can be different depending on experimental design and conditions, including SOC content, soil properties, BC type and dose, temperature, or soil moisture level. Usually, very simple experiments with only biochar and unfertilized soils are preferred; however, distinct effects can be observed when inorganic or organic fertilization is performed on biochar-amended soil. Nevertheless, literature reports stay in agreement that despite some initial contribution to CO_2_ emission from biochar-amended soils (mainly due to the rapid decomposition of the labile part), only a small part of charred biomass is bioavailable and the rest effectively contributes to the long-term carbon sink [45].

In our study, we hypothesized that labile organic matter (LOM) from cover crop residues or organic fertilizers, e.g., manures or compost, may change the C-sequestration potential in biochar-amended soil. Both types of C sources will contribute to the SOC priming effect and this may induce changes in the native mineralization process of organic matter, which in turn will increase or decrease CO_2_ flux from soil [46]. The results of the experiment showed that the introduction of EXOC to biochar-amended soil enhances CO_2_ fluxes, however not equally, and raw materials, e.g., cover crop residues will contribute to the process more actively than stable forms of organics like compost. The effect will also vary depending on soil type and properties. A more prominent stimulating effect of EXOC on CO_2_ emission was observed in sandy soil with biochar amendment. Faster BC-C mineralization in soil with low organic matter content is associated with good adaptation of microbes for limited nutrients and more effective utilization of available labile compounds in comparison with soils rich in native organic matter [1,2,14,47,48,49]. In terms of loamy soil, lower CO_2_ emissions can be explained by organo–mineral interactions and protection of organic matter against the mineralization process, which is claimed as a main factor of reduced GHG emission from soils with high clay mineral content [50,51].

Food waste is one of society’s highest volume and most environmentally impactful waste streams. Upcycling of food waste into usable materials can be integral to mitigate the substantial greenhouse gas emissions associated with wasted food [27]. Inference on the high stability of biochar in the soil environment is limited to a very narrow group of biochars produced from basic and generally available types of biomasses, and more attention should be paid to ‘new biochars’ obtained by utilizing household and food waste. As a very valuable source of nutrients and active compounds, its utilization as a soil amendment seems to be a natural way of waste upcycling. Although biochars from food waste can contribute to CO_2_ emissions, at least in the short-term, food wastes are produced in large amounts and their transformation to biochar seems to be a win–win solution in waste management and the upcycling of nutrients. Biochars are rich in nutrients and active components that can support plant growth and improve soil fertility [4,52]. Pyrolysis of biomass not only contributes to carbon sequestration but also allows the obtainment of organic fertilizer for sustainable agronomy [53]. Production of biochars closes the loop of waste management and supports circular economy strategies, as well as being able to reduce the emission of greenhouse gases, and regardless of biochar type, pyrolyzed biomass is more resistant to mineralization than easily decomposable agricultural residues and is able to lock carbon in soils. Nevertheless, there are some limitations that need to be addressed to make biochar production economically feasible [54]. Material drying and pre-treatment consumes energy and contributes to the process of CO_2_ emission, as well as generates costs [55]. This needs to be balanced, especially in the case of biochars that decompose the fastest (BC1—food waste or BC3—coffee grounds).

This work highlights the problem of future implications related to the incorporation of new types of black carbon into soil. Variability of soil CO_2_ fluxes in biochar-amended soils can be attributed to biochar and soil properties, but also inputs of exogenous organic matter from soil fertilization and other agronomic practices. The results of our study showed that feedstocks for biochar production are an important factor in determining the stability of the material in soil. Feedstock and soil properties’ effect on biochar stability should be taken into account in sustainable agriculture practices or climate change mitigation strategies.

## 5. Conclusions

The performed study confirms that biochars, when applied to the soil, are the subject of a slow mineralization process with CO_2_ release. The key factor that affects CO_2_ efflux from amended soil is the feedstock type used for biochar production, which determines further properties of biochar. CO_2_ efflux was the highest for food waste biochars containing more labile C fraction and that are consequently more susceptible to decomposition processes compared to high-cellulose biochar. The application of exogenous organic matter, especially raw organic plant residues and cow manure, to biochar-amended soils enhanced CO_2_ release and carbon losses; however, in the long term, the contribution of the process might be negligible. To predict biochar behavior in soil under different farming practices, it is necessary to develop field trials and provide data from long-term observation under natural conditions.

## Figures and Tables

**Table 1:** Summary of the treatments in the incubation experiment.

Description	Abbreviation	Dose Equivalent [t ha^−1^]
Sandy soil without amendments	SA	-
Sandy soil with six types of biochar	SA BC1—SA BC6 ^1^	0.57–0.92 (2% *v*/*w*)
Sandy soil with six types of biochar and three types of organic matter	SA BC1—BC6 CO for compost SA BC1—BC6 MA for manure SA BC1—BC6 LE for legumes	biochar: 0.57–0.92 (2% *v*/*w*) organics: 37.50 (1% *w*/*w*)
Silt loam soil without amendments	SiL	-
Silt loam soil with six types of biochar	SiL BC1—SiL BC6	0.57–0.92 (2% *v*/*w*)
Silt loam soil with six types of biochar and three types of organic matter	SiL BC1—BC6 CO for compost SiL BC1—BC6 MA for manure SiL BC1—BC6 LE for legumes	biochar: 0.57–0.92 (2% *v*/*w*) organics: 37.50 (1% *w*/*w*)

^1^—respectively, for all six biochar types.

**Table 2:** General properties of the soils, biochars, and organic amendments used in the experiment.

	Abbr. in Paper	Substrate			pH (H_2_O)	CEC ^1^ [cmol (+) kg^−1^]	TOC [g 100 g^−1^]	TN [g 100 g^−1^]	C:N	Ash [%]	CaCO_3_ [%]
Soils	SA	Loamy sand			4.62	1.62	0.72	0.04	16.9	n/a	0.25
		sand	silt	clay							
		[%]									
		81	17	2							
	SiL	Silt loam			6.40	11.70	0.99	0.07	13.7	n/a	0.00
		sand	silt	clay							
		[%]									
		22	64	15							
Biochars	BC1	Food wastes			9.41 ± 0.05	228	53.0 ± 1.10	0.98 ± 0.02	54.1	10.1 ± 1.00	n/a
	BC2	Cut green grass			10.43 ± 0.04	228	52.0 ± 1.00	2.70 ± 0.05	19.3	31.3 ± 3.10	n/a
	BC3	Coffee grounds			6.91 ± 0.07	35.0	68.0 ± 1.40	3.60 ± 0.07	18.9	3.70 ± 0.40	n/a
	BC4	Wheat straw			7.20 ± 0.13	7.41	76.0 ± 1.50	0.24 ± 0.05	317	1.30 ± 0.1	n/a
	BC5	Sunflower husks			10.29 ± 0.02	35.3	78.0 ± 1.60	0.63 ± 0.01	124	5.60 ± 0.60	n/a
	BC6	Beech wood chips			6.96 ± 0.07	22.7	70.0 ± 1.40	1.40 ± 0.03	50.0	9.80 ± 1.00	n/a
Organic matter	CO	Compost			5.66	10.8	17.6	2.01	8.77	n/a	n/a
	MA	Manure			7.00	n/a	28.0	4.00	7.00	n/a	n/a
	LE	Legume biomass			n/a	n/a	51.8	n/a	n/a	12.20	n/a

^1^ in table: Abbr. = abbreviation, CEC = cation exchange capacity, TOC = total organic carbon, TN = total nitrogen, n/a = not applicable. Values are means ± standard deviation (SD) from three replicates.

## Data Availability

Data sharing is not applicable to this article.

## Supplementary Materials

The following supporting information can be downloaded at: https://www.mdpi.com/article/10.3390/ma16216950/s1, Table S1: Carbon balance in incubated treatments.
