# Supplementary material for: Biochar and Organic Fertilizer Co-Application Enhances Soil Carbon Priming, Increasing CO2 Fluxes in Two Contrasting Arable Soils
Source: Materials (Basel). 2023 Oct 30;16(21):6950. doi: 10.3390/ma16216950 (PMC10649814; doi:10.3390/ma16216950)
Supplement: Supplementary file 1 [file materials-16-06950-s001.zip › materials-2642629-supplementary.pdf]

Supplementary data for the manuscript entitled "Biochar and Organic Fertilizer Co-Application Enhances Soil Carbon Priming Increasing CO_2 Fluxes in Two Contrasting Arable Soils"

Table S1. Carbon balance in incubated treatments.

**Treatment**	**C from soil** [g 100 g^{-1}]	**C from biochar** [g 100 g^{-1} soil]	**C from organic amendment** [g 100 g^{-1} soil]	**Total C content** [g 100g^{-1} soil]	**Total measured C-CO_2 loss** [g 100g^{-1} soil]	**Measured C loss in respiration** [%]
SA	0.721	n/a	n/a	0.721	0.0018	0.25
SA BC1	0.721	0.268	n/a	0.989	0.0100	1.01
SA BC2	0.721	0.210	n/a	0.931	0.0039	0.42
SA BC3	0.721	0.366	n/a	1.087	0.0057	0.52
SA BC4	0.721	0.350	n/a	1.071	0.0028	0.26
SA BC5	0.721	0.353	n/a	1.074	0.0023	0.22
SA BC6	0.721	0.512	n/a	1.233	0.0037	0.30
SiL	0.986	n/a	n/a	0.986	0.0021	0.21
SiL BC1	0.986	0.268	n/a	1.254	0.0065	0.52
SiL BC2	0.986	0.210	n/a	1.196	0.0031	0.26
SiL BC3	0.986	0.366	n/a	1.352	0.0064	0.47
SiL BC4	0.986	0.350	n/a	1.336	0.0035	0.26
SiL BC5	0.986	0.353	n/a	1.339	0.0028	0.21
SiL BC6	0.986	0.512	n/a	1.498	0.0036	0.24
SA + CO	0.721	n/a	0.176	0.90	0.0027	0.30
SA + MA	0.721	n/a	0.280	1.00	0.0051	0.51
SA + LE	0.721	n/a	0.163	0.88	0.0063	0.71
SA BC1 + CO	0.721	0.268	0.176	1.17	0.0085	0.73
SA BC1 + MA	0.721	0.268	0.280	1.27	0.0108	0.85
SA BC1 + LE	0.721	0.268	0.163	1.15	0.0127	1.10
SA BC2 + CO	0.721	0.210	0.176	1.11	0.0034	0.31
SA BC2 + MA	0.721	0.210	0.280	1.21	0.0059	0.49
SA BC2 + LE	0.721	0.210	0.163	1.09	0.0077	0.71
SA BC3 + CO	0.721	0.366	0.176	1.26	0.0068	0.54
SA BC3 + MA	0.721	0.366	0.280	1.37	0.0108	0.79
SA BC3 + LE	0.721	0.366	0.163	1.25	0.0093	0.75
SA BC4 + CO	0.721	0.350	0.176	1.25	0.0029	0.23
SA BC4 + MA	0.721	0.350	0.280	1.35	0.0059	0.44
SA BC4 + LE	0.721	0.350	0.163	1.23	0.0065	0.53
SA BC5 + CO	0.721	0.353	0.176	1.25	0.0031	0.25
SA BC5 + MA	0.721	0.353	0.280	1.35	0.0062	0.46
SA BC5 + LE	0.721	0.353	0.163	1.24	0.0076	0.61
SA BC6 + CO	0.721	0.512	0.176	1.41	0.0031	0.22
SA BC6 + MA	0.721	0.512	0.280	1.51	0.0098	0.65
SA BC6 + LE	0.721	0.512	0.163	1.40	0.0087	0.62
SiL + CO	0.986	n/a	0.176	1.162	0.0033	0.28
SiL + MA	0.986	n/a	0.280	1.266	0.0041	0.33

SiL + LE	0.986	n/a	0.163	1.149	0.0039	0.34
SiL BC1 + CO	0.986	0.268	0.176	1.430	0.0066	0.46
SiL BC1 + MA	0.986	0.268	0.280	1.534	0.0065	0.42
SiL BC1 + LE	0.986	0.268	0.163	1.417	0.0074	0.52
SiL BC2 + CO	0.986	0.210	0.176	1.372	0.0037	0.27
SiL BC2 + MA	0.986	0.210	0.280	1.476	0.0068	0.46
SiL BC2 + LE	0.986	0.210	0.163	1.358	0.0044	0.33
SiL BC3 + CO	0.986	0.366	0.176	1.528	0.0048	0.31
SiL BC3 + MA	0.986	0.366	0.280	1.632	0.0078	0.48
SiL BC3 + LE	0.986	0.366	0.163	1.514	0.0059	0.39
SiL BC4 + CO	0.986	0.350	0.176	1.512	0.0028	0.19
SiL BC4 + MA	0.986	0.350	0.280	1.616	0.0053	0.33
SiL BC4 + LE	0.986	0.350	0.163	1.498	0.0055	0.37
SiL BC5 + CO	0.986	0.353	0.176	1.515	0.0029	0.19
SiL BC5 + MA	0.986	0.353	0.280	1.619	0.0049	0.30
SiL BC5 + LE	0.986	0.353	0.163	1.501	0.0058	0.39
SiL BC6 + CO	0.986	0.512	0.176	1.675	0.0037	0.22
SiL BC6 + MA	0.986	0.512	0.280	1.778	0.0057	0.32
SiL BC6 + LE	0.986	0.512	0.163	1.661	0.0051	0.30

n/a = not applicable.
